# High levels of the openness trait are associated with better parental reflective functioning in mothers with substance use disorders

**DOI:** 10.1016/j.abrep.2020.100318

**Published:** 2020-11-12

**Authors:** Merete Glenne Øie, Ingebjørg Emilie Aarnes, Lise Horndalsveen Eilertsen, Kerstin Söderström, Eivind Ystrom, Ulrika Håkansson

**Affiliations:** a Department of Psychology, University of Oslo, Oslo, Norway; b Department of Research, Innlandet Hospital Trust, Lillehammer, Norway; c Inland Norway University of Applied Sciences, Lillehammer, Norway; d PROMENTA Research Center, Department of Psychology, University of Oslo, Oslo, Norway; e Department of Mental Disorders, Norwegian Institute of Public Health, Oslo, Norway; f Pharmacoepidemiology & Drug Safety Research Group, School of Pharmacy, University of Oslo, Oslo, Norway

**Keywords:** Parenting personality, Parental reflective function, Maternal, Substance abuse

## Abstract

**Aims:**

Mothers with substance use disorders (SUD) often show impairments in parental reflective functioning (PRF), which may have adverse effects on their capacity for sensitive caregiving. Parenting personality is also associated with caregiving. However, no studies have investigated how these individual factors may contribute to variance in PRF in mothers with SUD. In this study PRF and personality were assessed in 43 mothers with SUD.

**Methods:**

PRF was assessed by the Parent Development Interview. Personality traits were assessed by the Revised Neuroticism-Extraversion-Openness Personality Inventory.

**Results:**

The results indicate that higher levels of the Openness trait are associated with better PRF.

**Conclusion:**

Mothers low in Openness may need more specific and situational training in interpreting mental states in their children. Highly open mothers with SUD will likely need more help distinguishing the child’s mental states from their own, and might need help to maintain mutuality and regulating the intensity of their responses to the child’s behavior.

## Highlights

• Mothers with high levels of Openness had better parental reflective functioning.
• Mothers with low Openness need training in interpreting mental states in their children.
• It is important to assess the mothers personality before selecting form of treatment.

## 1 Introduction

As a group, mothers with substance use disorders (SUD) show difficulties understanding the meaning of their infant’s emotions and behavior, and in responding appropriately and sensitively to the children s cues (Suchman et al., 2011, De Falco et al., 2014). Reflective functioning (RF) is the observed manifestation of the ability to understand the mental state of others behind their overt behavior (i.e. mentalization) (Fonagy, Steele, Moran, Steele, & Higgitt, 1991). This crucial capacity is considered fundamental in sensitive caregiving, and thought to play an important role in the intergenerational transmission of attachment (Slade, 2005). Mothers with a SUD are often reported to have impairments in parental reflective functioning (PRF; Slade, 2005), although with individual variations (Håkansson et al., 2018a, Pajulo et al., 2006, Suchman et al., 2005).

Even though parenting practices have long been acknowledged as an expression of parents’ personality (Belsky, 1984), studies in the field of mentalization have suffered from a lack of consideration of individual differences in explaining variations of PRF (Sarfi et al., 2011, Luyten and Fonagy, 2015). However, high PRF in non-clinical samples have shown associations with caregiving behaviors like flexibility, responsiveness, curiosity and willingness to understand the child’s mental states (Fonagy et al., 1991, Fonagy et al., 2004, Luyten et al., 2017). Thus, personality factors may contribute to the variation in PRF among mothers with SUD. As far as we know, no studies have investigated how personality and personality traits may affect the capacity for PRF, and not in caregivers with SUD specifically, which is the focus of the current study.

The concept of personality has long been conceptualized from the perspective of The Five Factor Model (FFM; McCrae & Costa, 1987). The model emphasizes a hierarchical organization of the personality taxonomy, with five domains descriptive of behavior, each incorporating six more specific facets. Traits from the five personality domains measured by the FFM, i.e. Neuroticism (N), Extraversion (E), Openness to Experience (O), Agreeableness (A), and Conscientiousness (C), have also been linked to psychopathology and the vulnerability to substance abuse and risk-taking behavior (Rosenstrom et al., 2018, Kotov et al., 2010, Terracciano et al., 2008). For instance, high Neuroticism and low Conscientiousness are consistent patterns of the personality profiles of people with SUD (Kotov et al., 2010).

Some research has investigated the relationships between the five personality domains measured by the FFM and sensitive caregiving in non-clinical mothers. Bornstein et al. (2011) described Openness as a positive parenting trait, related to mothers' parenting knowledge, and their reported competence and investment in parenting. A meta-analysis of Prinzie et al. (2009) found the Extraversion trait to be crucial in predicting parenting warmth. This outcome often refers to the extent to which parents intentionally foster individuality, self-regulation and support, by being attuned and indulgent to the child’s demands. Coplan et al. (2009) also found Agreeableness to be positively related to parental warmth, responsiveness and authoritative parenting in general, as well as promoting more positive emotion regulation in their children. On the other hand, the same researchers found Neuroticism to be most negatively associated to warm and responsive parenting.

*Aims and hypotheses*

The main objective in the present study was to investigate how personality traits and their sub facets measured by the FFM relate to PRF in a sample of mothers with SUD. The results may highlight the relevance of the personality assessment in clinical practices and help identify possible areas of intervention in order to improve the interaction between these mothers and their children. This may also positively affect the children’s development of own reflective functions. We hypothesized that in a group of mothers with SUD, high levels of the Agreeableness, Openness and Extraversion traits were associated with better PRF. Further, we predicted higher levels of Neuroticism to be associated with lower PRF.

## 2 Methods

### 2.1 Sample and procedure

The data used in this study was cross-sectional, and collected as part of a larger study (Håkansson et al., 2018a, Håkansson et al., 2018b). The purpose of the larger project was to generate knowledge about, and promote well-being for children residing in families with parental substance abuse and/ or parental mental illness. Only selected data are included and presented in the current study because results from the other methods are reported in the other publications. Full assessment was conducted in the participants’ homes or in the treatment facilities where they had been living for the last year during 3–6 sessions. In total, the assessment took approximately seven hours. A clinical psychologist collected all the data, supervised by a specialist in clinical neuropsychology.

Our study is based on data on 43 mothers who completed the entire assessment battery in the larger study. The recruitment period lasted for two years. The inclusion criteria were a former substance use problem and a current SUD diagnosis, with or without a comorbid mental illness. The mothers reported to be abstinent during the assessment period. The exclusion criteria were an estimated full-scale IQ below 70 in the mothers, multiple pregnancy, premature birth (<32 weeks and 1500 g), or a severely ill or multi-handicapped child.

The project was approved by The Norwegian Regional Committee for Medical Research Ethics in Eastern Norway (REK-Ost, Nr. 2012/1370). The research was conducted in accordance with the Helsinki Declaration of the World Medical Assembly.

### 2.2 Measures

#### 2.2.1 Substance use and mental disorders

We used the Norwegian version of the fifth edition of The European Severity Index (Europ-ASI; McLelland et al., 1992) to register use of psychoactive substances. All diagnoses were based on the International Classification of Mental and Behavioural Disorders (ICD-10; World Health Organization [WHO], 1992). Comorbid psychopathology were screened for with the Norwegian version of the Mini-International Neuropsychiatric Interview 5.0.0 manual (M.I.N.I; Sheehan et al., 1998).

#### 2.2.2 Parental reflective functioning

The Parent Development Interview revised was used to assess reflective functioning (PDI-R2; Slade et al., 2005a, Aber et al., 1985, Fonagy et al., 1998). PDI-R2 is a semi-structured clinical interview intended to investigate a parent’s ability to think reflectively about their child, themselves in the parent role, and their relationship with their child. The interview was recorded and transcribed from audio files and coded by an independent coder in accordance with evaluation guidelines developed by Fonagy et al. (1998). A second coder coded 25% of the interview to ensure stronger inter-rater reliability. There was a strong intra-class correlation between the coders (r = 0.96). The validity of the PDI-R2 has been found satisfactory in populations with substance abusing mothers (Slade, 2005). In the current study, the Cronbach *α* coefficient for PDI-RF2 scale was 0.88, indicating a good internal consistency. The interviews were scored on an 11-point scale from −1 to 9, i.e., organized along a continuum from low to high RF. In the scoring manual, a score of −1 implies negative RF, and includes responses characterized as distinctively anti-reflective, bizarre or inappropriate in the context of the interview. A score of 9 indicates exceptional RF, with complex, elaborating, sophisticated and surprising reflections. A score of 5 is termed definite or ordinary RF, involving some elements that makes the reflection explicit (Slade et al., 2005a). Mothers having a total PRF score of 3 or lower are defined as having a negative or low PRF (See also Håkansson et al., 2018a, Håkansson et al., 2018b).

#### 2.2.3 Five factor personality

Personality was assessed using the Norwegian version of the Revised NEO Personality Inventory (NEO PI-R; Costa and McCrae, 2008, Martinsen et al., 2003). The NEO PI-R is a self-report questionnaire with 240 items, developed to measure the five major domains of personality described in the Five Factor Model of personality: Neuroticism (N), Extraversion (E), Openness to Experience (O), Agreeableness (A), and Conscientiousness (C). Each factor consists of six more specific facets. Respondents answer on a five-point Likert scale, ranging from strongly agree to strongly disagree. The NEO PI-R scores were standardized to t-scores using Norwegian norms. The NEO PI-R profile marks five T-score levels: *very low* (20–34), *low* (35–44), *average* (45–55), *high* (56–65), *very high* (66–80) (Costa & McCrae, 2008). Reliability for the NEO-PI-R has been reported to be satisfactory (Costa and McCrae, 1995, Källmen et al., 2011), also in a Norwegian clinical population (Østbø & Nordvik, 2008)*.* For our sample of mothers, the mean Cronbach α coefficient for the NEO-PI-R facets was 0.75, indicating satisfactory internal consistency for the scale.

### 2.3 Statistical analyses

All cases (*N* = 43) were included in the analyses, and there was no missing data. Associations between personality domains and facets and PRF were estimated using a Pearson correlation coefficient analysis. In order to account for multiple testing, we Bonferroni corrected the alpha value 0.05:35 = 0.001. Standardization of t-scores from the NEO PI-R was conducted using the general mean and standard deviations from the SUD population in particular because our sample is selected and expected to have clinically low scores on the PDI-R2 (*M* = 50, *SD* = 10). The process of computing means and standard deviations from this norm population was based on the research of Stacks et al. ([*M* = 4.57, *SD* = 1.47], 2014), Pajulo et al. ([*M* = 3.1, *SD* = 1.00], 2008; [*M* = 3.00, *SD* = 1.00], 2012) and Suchman et al. ([*M* = 3.1, *SD* = 0.5], 2017). These researchers studied PRF in a total of 209 women with SUD. The mean PRF scores derived from these studies constituted an average PRF score in the population of *M* = 3.4 (*SD* = 1.0). These values were set as the standard of reference in our sample when transforming raw scores of the PDI-R2 to standard scores.

## 3 Results

### 3.1 Sample characteristics

The characteristics of the study sample are summarized in Table 1. Twelve of the mothers (27.9%) were recruited from outpatient clinics, and six mothers (14.0%) were recruited by health nurses working in nearby municipalities. The remaining 25 mothers (58.1%) were recruited from treatment facilities specialized in caring for pregnant women and families with small children, with a concurrent substance abuse problem.

**Table 1:** Demographic data.

	Range	Mean (SD)	
Mother’s age	19–44	31.07 (6.37)	
Child’s age (months)	1–18	8.56 (3.79)	
Number of children	1–4	1.51 (.80)	
Children in daily custody	0–2	1.00 (.22)	
Longest continuous period of work (months)	1–132	31.07 (31.78)	
Civil status:	Number	Percentage	
Cohabitant	14	32.6	
Romantic partner	7	16.3	
Single	22	51.2	
*Highest completed education:*			
Did not complete Primary school	2	4.7	
Primary school	23	53.5	
High school	12	27.9	
Graduate degree	4	9.3	
Master/professional degree	2	4.7	
*Mental health data*a)	Number	Percentage	
ADHD	4	9.3	
Current depression	16	37.2	
Previous depression	41	95.3	
Previous suicide attempt	29	67.4	
Self-harm	28	65.1	
Mani	16	37.2	
Bipolar	2	4.7	
Panic	26	60.5	
Agoraphobia	12	27.9	
Social phobia	21	48.8	
Obsession	11	25.6	
Compulsion	5	11.6	
OCD	1	2.3	
PTSD	29	67.4	
General anxiety	23	53.5	
Psychosis	18	41.9	
Drug induced psychosis	22	51.2	
Schizophrenia	0	0.0	
Anorexia	16	37.2	
Bulimia	8	18.6	
Binge eating	4	9.3	
*Somatic health data, mother*			
Chronic disease	4	9.3	
Hepatitis	17	39.5	
HIV	2	4.7	
*Substance abuse mother*b)	Preferred %	Mean debut age (SD)	Problematic % (N = 43)
Alcohol	16.3	13.09(2.98) (n = 42)	41.9
Abuse of prescribed medications	0.0	18.08(5.79) (n = 37)	74.4
Cannabis	14.0	16.21(4.39) (n = 42)	81.4
Amfetamin/Cocaine	37.2	17.82(4.42) (n = 38)	72.1
Opiates	32.6	20.28(5.95) (n = 25)	46.5
Poly-substance use	0.0	18.36(4.78) (n = 36)	74.4

*Note.* N = 43 SD = Standard deviation.

a) Mini-International Neuropsychiatric Interview 5.0.0 manual

b) European Addiction Severity Index (Europ-ASI) 5th edition.

Children with neonatal abstinence syndrome (NAS) were not excluded, and eleven babies (25.6%) were born with the diagnosis. The offspring sample consisted of 15 girls (34.9%), and 28 boys (65.1%), with mean age 8.6 months (SD = 3.8, range 1–18 months). The majority of mothers (62.8%) were primipara. During the inclusion period, 12 of the mothers (27.9%) lost custody of the child participating in the study.

### 3.2 Descriptive statistics of the PDI-R2 and the NEO-variables

Scores on the PDI-R2 indicated an average poor total PRF for the group (mean score was 2.91, *SD* = 1.17), as 74.4% of the mothers scored at the cut-off point (e.g. 3) or lower. The variability in total PRF scores were moderate, extending from 0 to 6.

The descriptive statistics of the NEO-variables are presented in Table 2. The sample mean of Neuroticism (N) was in the “high” area compared to a normative sample. Extraversion (E) and Conscientiousness (C) was in the “low” area of the normal distribution. Openness (O) and Agreeableness (A) were both in the “average” area.

**Table 2:** Personality profile descriptives.

Personality profile a)	Range	Mean (SD)	t-score intervals b)
**Neuroticism (N)**	33–82	61.84 (9.57)	**HIGH**
Anxiety (N1)	42–78	60.47 (8.43)	
Hostility (N2)	22–94	58.33 (12.88)	
Depression (N3)	43–80	60.70 (8.59)	
Self-consciousness (N4)	38–82	61.40 (10.24)	
Impulsiveness (N5)	27–75	49.33 (10.73)	
Vulnerability (N6)	33–82	61.72 (12.36)	

**Extraversion (E)**	15–76	40.33 (13.05)	**LOW**
Warmth (E1)	24–68	47.09 (11.36)	
Gregariousness (E2)	15–65	38.37 (10.73)	
Assertiveness (E3)	24–67	42.77 (11.22)	
Activity (E4)	26–67	44.65 (10.05)	
Excitement-seeking (E5)	24–66	45.47 (9.03)	
Positive Emotion (E6)	13–71	42.35 (14.81)	

**Openness (O)**	32–71	46.26 (9.57)	**AVERAGE**
Fantasy (O1)	31–68	47.30 (9.53)	
Aesthetics (O2)	16–70	49.26 (11.05)	
Feelings (O3)	33–75	49.07 (10.35)	
Actions (O4)	17–61	44.77 (8.09)	
Ideas (O5)	33–65	47.37 (10.02)	
Values (O6)	31–71	46.53 (8.58)	

**Agreeableness (A)**	14–79	48.88 (12.78)	**AVERAGE**
Trust (A1)	9–74	39.26 (14.45)	
Straightforwardness (A2)	25–72	50.51 (11.22)	
Altruism (A3)	17–70	50.53 (13.22)	
Compliance (A4)	12–76	50.53 (13.03)	
Modesty (A5)	36–71	53.05 (9.69)	
Tender-mindedness (A6)	23–74	51.26 (10.39)	

**Conscientiousness (C)**	17–69	43.00 (11.97)	**LOW**
Competence (C1)	13–74	41.58 (13.68)	
Order (C2)	30–70	47.47 (9.37)	
Dutifulness (C3)	17–69	45.47 (11.47)	
Achievement striving (C4)	31–67	45.49 (9.43)	
Self-discipline (C5)	20–69	41.88 (12.42)	
Deliberation (C6)	16–68	46.44 (11.13)	

*Note*. N = 43 SD = Standard deviation,

a) Revised NEO Personality Inventory (NEO PI-R). Values are reported in t-scores,

b) t-score intervals (See Costa & McCrae, 2008).

### 3.3 Associations between personality factors and facets and PRF

The Openness-factor was the only broad personality trait significantly associated with the PRF. There was a moderate, positive association between the Openness-factor and PRF (*r* = 0.44), with higher levels of Openness being associated with better PRF. The facets of Openness which were associated with PRF, were O1: Fantasy (*r* = 0.35), O3: Feelings (*r* = 0.43) and O5: Ideas (*r* = 0.40). The facet N2: Hostility, associated with the Neuroticism-factor, was negatively and moderately correlated to PRF (*r* = -0.34). A6: Tender-mindedness, was associated with better PRF (*r* = 0.36)*.* The facet C4: Achievement-striving was associated with better PRF (*r* = 0.34). See Table 3.

**Table 3:** Pearson correlation coefficients between personality factors and facets and PRF.

Personality factors/ facets ^a)^	PRF ^b)^
**Neuroticism (N)**	−0.26
Anxiety (N1)	−0.15
Hostility (N2)	−0.34*
Depression (N3)	−0.24
Self-consciousness(N4)	0.09
Impulsiveness (N5)	−0.16
Vulnerability (N6)	−0.26
**Extraversion (E)**	0.24
Warmth (E1)	0.29
Gregariousness (E2)	0.07
Assertiveness (E3)	0.23
Activity (E4)	0.17
Excitement-seeking (E5)	−0.06
Positive emotions(E6)	0.29
**Openness (O)**	0.44**
Fantasy (O1)	0.35*
Aesthetics (O2)	0.15
Feelings (O3)	0.43**
Actions (O4)	0.02
Ideas (O5)	0.40**
Values (O6)	0.24
**Agreeableness (A)**	0.22
Trust (A1)	0.01
Straightforwardness (A2)	−0.05
Altruism (A3)	0.26
Compliance (A4)	0.23
Modesty (A5)	0.15
Tender-mindedness (A6)	0.36*
**Conscientiousness (C)**	0.26
Competence (C1)	0.29
Order (C2)	0.19
Dutifulness (C3)	0.20
Achievement striving (C4)	0.34*
Self-discipline (C5)	0.24
Deliberation (C6)	0.15

## 4 Discussion

We found that higher levels of the Openness trait are associated with better PRF, including the associated facets Fantasy, Feelings and Ideas, and PRF in our sample of mothers with SUD. This relationship, i.e. that Openness is highly related to PRF, is supported empirically and theoretically; Openness broadly reflects an individual’s receptiveness to new experiences, both internally (exploring internal emotions and ideas) and externally (exposing oneself to new, unfamiliar things). Openness distinguishes between those who seek out novelty and avoid structure/rules, and those who seek stability and familiarity. Arguably, how well a person is able to balance external pressures for change, in relation to internal emotional drives, will most likely affect the capacity for adjustment (Piedmont, Sherman, & Sherman, 2012); for instance in relation to one’s child. In light of our results, it is plausible that the level of receptiveness to novel features in the child’s behavior, as they spontaneously and unexpectedly occur in interaction, is important for the mother's capacity to interpret the child’s underlying mental states.

Low Openness is associated with personal and social adjustment problems, restricted interests, low tolerance for differing perspectives, and inability to understand and express own feelings. Low levels, especially the facets Feelings and Ideas, are also empirically related to Alexithymia (Widiger, Costa, & McCrae, 2002). This trait is characterized by difficulties in identifying and describing subjective feelings, limited imagination, and an externally orientated cognitive style (Taylor & Bagby, 2013). Alexithymia is also prevalent among persons with SUD (Taylor, Bagby, Kushner, Benoit, & Atkinson, 2014). Low Openness is thought to involve alienation and over-regulation of internal experiences (rigidity), over-reliance on external demands, reduced spontaneity and detection of one’s true feelings (Piedmont et al., 2012). A parent with a very low Openness-score will likely struggle to tolerate and adjust in situations where the child has different needs and perspectives, which is considered an important indicator of PRF (Slade et al., 2005a, Slade et al., 2005b). This raises the hypothesis that Openness may affect the ability to identify and regulate own mental states in relation to the child. Arguably, this supports previous findings indicating that impaired self-mentalizing may be particularly critical for PRF and parental sensitivity among mothers with SUD (Suchman, Decoste, Leigh, & Borelli, 2010).

Of relevance to our findings, Sobkow, Traczyk, Kaufman, and Nosal (2018) found that the Openness facets Fantasy and Ideas positively predicted a preference for using intuition. Thus, Openness seems to guide processing of internal and external information that may be important for PRF. Parents who detect more cues from their child’s behavior, and combine these more flexibly, will naturally be able to interpret and re-represent (“mirror”) underlying needs and intentions more accurately. Furthermore, these characteristics of high Openness may likely function as a buffer against breakdown in controlled, reflective mentalizing during affect-laden moments with the child. Very high Openness-scores may also contain dysfunctional aspects and affect PRF negatively. Highly open individuals are found to become overly absorbed in own ideas, fantasies, and eccentric thinking, tend to have a diffuse identity and unstable goals, and demonstrate non-conformity (Piedmont et al., 2012). A parent with highly permeable boundaries, marked by emotional intensity and unclear distinctions between self and others, may for example struggle to distinguish between the mental states of the child and one’s own, which in turn might lead to intrusiveness and impaired mutuality in the dyad. We did not investigate such a curvilinear association between Openness and PRF in this limited sample. This may be of interest for future research.

In discordance with our hypotheses, we did not find the domains of Extraversion, Agreeableness or Neuroticism to be associated with PRF in this limited sample. One possible explanation is that most of the involved facets of Extraversion, Agreeableness and Neuroticism do not adequately underpin the crucial reflective component of PRF, as measured by the PDI-R2. Quality of PRF depends on the parent’s capacity to both reflect upon and re-represent the child’s mental states accurately (Kelly, Slade, & Grienenberger, 2005). Given the inherent characteristics of these three personality domains, it is likely that they are more related to the more global construct of parental sensitivity. However, our results show that the N2-facet Hostility, the A6-facet Tender-mindedness, and the C4-facet Achievement striving, were all able to predict PRF.

In terms of N2: Hostility, this facet measures the tendency to experience anger (Martinsen et al., 2003). We suggest that mothers with higher N2-scores may experience the emotional demands of the baby as more overwhelming, and show propensity to attribute too much negativity into the infant’s cues. As a result, the mother’s (mis)perception of the baby is reflecting a lower PRF score. In the parenting literature, high scores on the A6-facet is related to parents who are better able to identify and respond to their child’s needs (Le Vigouroux, Scola, Raes, Mikolajczak, & Roskam, 2017). We suggest that mothers with high A6-scores may be apt at mentalization, which likely improves PRF. Furthermore, the Agreeableness-trait in general is also positively associated with social support seeking, active coping, planning, and positive reappraisals, when encountering a distressful experience (Afshar et al., 2015). The Conscientiousness-factor in general is thought to operate as a protector of substance dependency (Raketic et al., 2017). Therefore, SUD-mothers characterized as having higher C4 Achievement striving-scores may be more successful in staying abstinent from drugs.

In previous research on the same group of mothers we have reported that mothers with negative to low PRF had significantly more experiences of adversities in early childhood and latency, and more impairments in executive functions, compared to the mothers with moderate to high PRF (Håkansson et al., 2018a, Håkansson et al., 2018b). Thus, both trauma and impaired cognitive functions may also be associated with PRF. PRF involves an ability to appreciate the inner world of oneself and others. It has been hypothesized that early adversity in attachment relationships leads to a ‘fear of minds’, and difficulties with boundaries between self and others (Fonagy, 2010). Executive functions are higher-level cognitive functioning involved in the control and regulation of lower-level functioning such as emotional and behavioral processes (Miller & Cohen, 2001). A consequence of poor executive functioning is dysregulated affect and behavior, which in turn are associated with the inability to accurately mentalize about the inner world of the child (Fewell, 2010). Further, different drugs and comorbid psychopathology may also have had different effects on the mothers RF. In our previous study on the same group of mothers, we found that using multiple substances had negative associations with PRF, but not preference of a specific type of substance (Håkansson et al., 2018a). Substance abuse can cause alterations in biological processes and responses to infant sensory stimuli, which may lead to inappropriate maternal behavior (Newman et al., 2011, Rutherford et al., 2013). We also found that reduced general mental health status measured with the Hopkins Symptom Checklist (HSCL-10) showed a significant negative association with the mothers RF (Håkansson et al., 2018a).

**Strengths and limitations**

This study is the first to conduct an in-depth inspection of personality structure in predicting the capacity for PRF, using a high-risk group that is commonly hard to reach to research. We had no missing data and well validated and valid measurement tools, including semi-structured interviews.

Despite the strengths of our study, several limitations may have implications for the generalizability of our findings: Our relatively small sample size restrained the study, and the subjects were heterogeneous in regard to high rates of current and past psychopathology and substance use. Because of the relatively small sample size we could not do separate analyses on subgroups regarding different drugs used or comorbid diagnoses. Not considering the possible impact of different drugs or other diagnoses may be considered as a limitation of the present study. However, most of the mothers used multiple drugs and their mental health were characterized by comorbidity. This is common in individuals with SUD (Connor et al., 2014, Najt et al., 2011, Schulte and Hser, 2013). Thus, we consider our group of SUD mothers to be representative. Another limitation is that because the associations between PRF and personality not have been investigated in mothers without SUD, we do not know if our results are specific for mothers with SUD or not.

As expected from previous research on mothers with a SUD, a majority of the mothers scored below cut-off on the PDI-R2. Although the results are likely to be representative for mothers with SUD, it might have created methodological and interpretative limitations. Limitations in variability in this sample of mothers limits the possibility to draw conclusions concerning healthy mothers or mothers from other clinical populations. Also, selection bias may have occurred in the inclusion phase. For instance, mothers with SUD who are not seeking treatment may often exhibit more risk factors and less motivation towards improving the relationship with their children (Zilberman & Blum, 2005). However, the reader should keep in mind that we are studying PRF and personality in mothers with SUD specifically, and not in the population of mothers in general.

Further, based on our theoretical focus, we have tested one model regarding possible associations. However, no single model can fully predict reality. Our model is one out of many possible approaches, and other models may be plausible. For example one could conclude that better PRF causes more openness. It is also difficult to distinguish different models in a cross-sectional study, i.e., data is sampled at one point in time. Hence, longitudinal research might be valuable in the future.

The lack of screening for personality disorders is a limitation. We are aware that there is a high prevalence of such diagnoses in populations of substance abusing individuals (Casadio et al., 2014). Borderline and Antisocial personality disorders are particularly associated with comorbid addiction (Kienast et al., 2014, Goodwin and Hamilton, 2003), hence we expected personality disorders to influence the average personality profile and the PRF scores of our sample. This because most mental disorders, including personality pathology in particular, often involve difficulties with mentalizing (Bateman & Fonagy, 2010). Interestingly, whereas Neuroticism and Extraversion are strongly linked to personality disorders (Widiger et al., 2002), Openness shows no significant relations to any of them on a meta-analytical level (Samuel & Widiger, 2008). This may support the notion that Openness is a robust predictor of PRF, possibly less affected by psychopathology (Rosenstrom et al., 2018). The non-significant associations between the factors Extraversion, Agreeableness and Neuroticism the PRF score, may reflect limitations in our data rather than real lack of associations.

**Clinical implications**

Our research suggests that the Openness-trait is an especially important factor underlying individual variations of PRF in mothers with SUD. Individuals who are very low in Openness, might need a more comprehensive and lengthy mentalization-based treatment to develop and sensitize the parent to (own and others’) mental states. Low Openness may indicate a need for more specific and situational training in interpreting mental states. As described, very high Openness is also associated with adaptive problems. Highly open parents will likely need more help distinguishing the child’s mental states from their own, and might need help to maintain mutuality and with regulating the intensity of their responses to the child’s behavior.

The Five Factor dimensions might prove useful in clinical practice (Widiger & Presnall, 2013). This personality assessment provide a thorough and respectful understanding of the patients’ strengths and vulnerabilities. The FFM of personality recognizes that despite the presence of some maladaptive personality traits, other aspects of the self can be highly adaptive, depending on the situation (Widiger et al., 2002). Furthermore, so called personalized therapy is increasingly popular, and selecting the optimal form of treatment for each patient could be accomplished using the FFM of personality as a treatment guideline. For instance, high Openness may indicate an interest in exploratory psychotherapy, high Agreeableness may predict engagement in group therapy, and high Conscientiousness could indicate a willingness to accomplish, and an ability to appreciate the demands and stringency of dialectical behavior therapy (Widiger & Mullins-Sweatt, 2009). Interestingly, Fonagy and Allison (2014) demonstrated that treatments designed to increase RF also promoted personality changes. They argued that mentalization-based approaches to therapy may help patients with personality pathology to reduce rigidity, adjust behavior and actions, and improve their understanding of social relationships. Thus, in order to select the optimal form of treatment of mothers with SUD, it may be important to get more knowledge about the mothers personality.

## CRediT authorship contribution statement

**MØ:** Conceptualization, Data curation, Supervision, Writing - original draft, Writing - review & editing. **IEA and LHE:** Conceptualization, Methodology, Formal analysis, Visualization, Writing - original draft, Writing - review & editing. **KS:** Funding acquisition, Conceptualization, Writing - review & editing. **EY:** Methodology, Supervision, Formal analysis, Writing - review & editing. **UH:** Conceptualization, Project administration, Data curation, Investigation, Methodology, Writing - review & editing.

## Role of Funding Sources

The project received financial support from The Research Council of Norway (RCN), grant number 213079/H10. EY was supported by RCN grant number 288083.

## Declaration of Competing Interest

None.
